# Measuring coverage of infant and young child feeding counselling interventions: A framework and empirical considerations for survey question design

**DOI:** 10.1111/mcn.13001

**Published:** 2020-04-15

**Authors:** Jowel Choufani, Sunny S. Kim, Phuong Hong Nguyen, Rebecca Heidkamp, Laurence Grummer‐Strawn, Kuntal Kumar Saha, Chika Hayashi, Vrinda Mehra, Silvia Alayon, Purnima Menon

**Affiliations:** ^1^ International Food Policy Research Institute Washington District of Columbia USA; ^2^ Johns Hopkins Bloomberg School of Public Health Johns Hopkins University Baltimore Maryland USA; ^3^ Department of Nutrition and Food Safety World Health Organization Geneva Switzerland; ^4^ Data and Analytics UNICEF New York USA; ^5^ Save the Children Washington District of Columbia USA

**Keywords:** coverage measurement, infant and young child feeding, nutrition counselling

## Abstract

Most countries implement nutrition counselling interventions as part of programmes to support breastfeeding and complementary feeding. However, data to track coverage of counselling interventions are rarely available. As a result, little is known about the coverage of counselling on infant and young child feeding (IYCF). Survey‐based data collection systems generally collect data on IYCF practices but do not collect data on coverage of interventions to support IYCF, and those surveys that do collect this information do not do so consistently. We present a framework to guide the design of survey questions to measure IYCF counselling coverage. We provide examples of how large‐scale surveys for programme evaluation and national monitoring have included survey questions to address these data gaps. Our review suggests that elements relevant to designing survey questions to capture coverage of counselling interventions include timing of contact, target behaviour and message content, place of contact, type of service provider, frequency of contact and mode of intervention. Application of this framework may help strengthen harmonized measurement of IYCF counselling coverage to enable better tracking of programme investments, document progress in scaling up nutrition services and allow for cross‐country comparisons. Thus, improving measurement of counselling coverage may lead to improved reach of programmes to support optimal IYCF practices.

**Key messages**

- Data to track coverage of counselling interventions are unavailable, so little is known about the coverage of counselling on infant and young child feeding.
- We propose a framework on six elements relevant to designing survey questions to capture coverage of counselling interventions: (1) timing of contact, (2) target behaviour and message content, (3) place of contact, (4) type of service provider, (5) frequency of contact and (6) mode of intervention.
- Application of the proposed framework can help to harmonize IYCF counselling coverage measurement and indicator development. This in turn can allow for better tracking of progress towards providing caregivers of infants and young children with age‐appropriate counselling services.

## 1 INTRODUCTION

Counselling is an effective behaviour change intervention for improving breastfeeding and complementary feeding (Bhutta et al., 2008; Dewey & Adu‐Afarwuah, 2008; Fabrizio, van Liere, & Pelto, 2014; Imdad, Yakoob, & Bhutta, 2011; Shi & Zhang, 2011; Sinha et al., 2015). Global evidence is also accumulating on the impact of integrating counselling interventions into health systems and large‐scale community‐based programmes (Menon et al., 2016a; Menon et al., 2016b). In the Global Nutrition Policy Review by the World Health Organization (WHO), a majority of countries surveyed report that they implement programmes to support breastfeeding and complementary feeding (World Health Organization, 2018a).

Despite this evidence base and the expanding global scale of programmes that include actions such as counselling to support breastfeeding and complementary feeding, little is known about the coverage of these programmes at a population level (Gillespie et al., 2019). Most survey‐based data collection systems do not include standardized indicators to capture the coverage of interventions that promote infant and young child feeding (IYCF). Only recently, the Demographic and Health Survey (DHS) Program updated its questionnaires for DHS‐8 to include questions on coverage of nutrition counselling during pregnancy, during antenatal care (ANC) and for infants (The DHS Program, 2019). Until this new data are available and due to the lack of coverage data, global monitoring initiatives such as the Countdown to 2030 and the Global Nutrition Report have traditionally used compiled survey data on IYCF practices (UNICEF, 2019) as proxies for programme performance. This raises several challenges because indicators of practices do not accurately reflect intervention coverage; IYCF practices are affected by several factors (such as knowledge, cultural norms and access to resources) and are therefore a poor proxy for intervention coverage. At the same time, it is apparent that the availability of standard indicators for assessing IYCF practices, defined by WHO and UNICEF in 2008 (World Health Organization, 2008, 2010) has advanced the ability to collect harmonized IYCF‐related data. The availability of these indicators has in turn allowed countries to identify gaps in child feeding practices, make cross‐country comparisons and begin to evaluate the progress of IYCF programmes. It has also spurred action to invest in programmes to strengthen these practices globally. The power of measurement is apparent in this example, and similar information on the reach of programme efforts being rolled out globally is needed.

Counselling interventions to improve IYCF must be tailored to context but must also include some common core elements and approaches. Key elements of intervention design include the counselling content, types of service providers, location of services and the timing and frequency of contact (McCarthy et al., 2016). In 2018, WHO released a guideline to provide global, evidence‐informed recommendations on breastfeeding counselling as a public health intervention to improve breastfeeding practices (World Health Organization, 2018b). The guideline recognized these design elements and made recommendations on some elements of breastfeeding counselling such as frequency, timing, mode and provider.

A technical consultation on IYCF counselling coverage measurement was held in September 2018 (Alive, & Thrive, DataDENT, IFPRI, UNICEF, & WHO, 2018), where technical experts from international and bilateral agencies and non‐governmental organizations discussed development of indicators to measure coverage of IYCF counselling or behaviour change interventions. During the consultation, the various elements for measuring IYCF counselling coverage were discussed, and one outcome was a minimum set of questions for inclusion in ANC, postnatal care (PNC) and child health and nutrition modules of household surveys. These questions were intended to enable collection of harmonized data on key elements of IYCF counselling coverage (Alive & Thrive et al., 2018) (Table S1).

In this paper, we present a framework describing all the different elements for measuring coverage of IYCF counselling and provide examples from large‐scale household surveys of how these elements are operationalized in survey questions. We present findings from a review of research protocols and questionnaires used in various evaluation studies and discuss considerations drawing from the broader literature on health service coverage measurement. The aim of this paper is to provide suggestions for a way forward for developing indicators to measure coverage of IYCF counselling.

## 2 METHODS FOR DEVELOPING THE FRAMEWORK AND ITS ELEMENTS

We conducted a review of research protocols and household survey questionnaires from impact evaluation studies and large‐scale nationally representative surveys in the previous 5 years (2013–2018) that measured coverage of IYCF counselling through interpersonal communication interventions. A total of 15 programme evaluations and three nationally representative surveys were reviewed (Table S2). Twelve programme evaluations included interventions that focused either on IYCF specifically, or overall maternal nutrition or prevention of undernutrition with IYCF as a component, and were implemented in various countries in South Asia and Africa. The three nationally representative surveys included Performance Monitoring and Accountability (PMA2020), DHS and Multiple Indicator Cluster Surveys (MICS) in various countries. We contacted the principal investigators directly to understand their process in measuring intervention coverage and to obtain their study documents. We reviewed all relevant study documents and consolidated survey questions into a database and analysed them for common and unique patterns based on multiple dimensions related to the interventions.

## 3 DEFINING MULTIDIMENSIONALITY OF COUNSELLING COVERAGE

**FIGURE 1:**
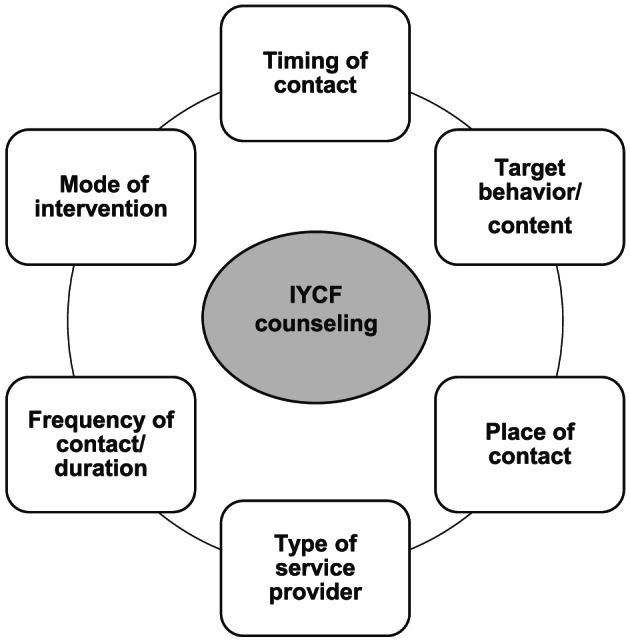
Six elements of IYCF counselling coverage questions. IYCF, infant and young child feeding

In general, we defined IYCF counselling coverage as the proportion of the target population such as mothers with children less than 2 years of age who received counselling about breastfeeding and complementary feeding. Given the multidimensionality of IYCF counselling interventions, however, we went further to identify six dimensions of IYCF counselling coverage from the literature and our database of survey questions—timing, target behaviour/content, place of contact, type of service provider, frequency and mode of intervention (Figure 1). Our framework builds on Tanahashi's seminal work on health service coverage, which identified five dimensions of effective coverage: availability, accessibility, acceptability, contact and effectiveness (Tanahashi, 1978). Whereas Tanahashi's model cascades from service capacity to the effectiveness or impacts of health interventions among beneficiaries, our coverage measurement framework proposes elements that capture all aspects of IYCF counselling interventions themselves. We do not address the measurement of counselling effectiveness in this paper. In this section, we describe each dimension and provide examples of questions and empirical considerations that reflect these elements from a large‐scale programme evaluation [Box 1: Alive & Thrive (A&T) Bangladesh] and a nationally representative survey (Box 2: PMA2020 Survey).

**BOX 1:** Alive & Thrive Bangladesh IYCF programme

Alive & Thrive is a multicountry nutrition initiative to save lives, prevent illness and ensure healthy growth of mothers and children. In Bangladesh, BRAC, a large national non‐governmental organization, delivered standard (non‐intensive) and intensified interpersonal counselling (IPC) and community mobilization (CM) as well as a mass media (MM) campaign (intensive) in 50 rural subdistricts in Bangladesh. For standard nutrition IPC, BRAC frontline health workers (Shasthya Kormi) and community health volunteers (Shasthya Shebika) conducted routine home visits and provided information on IYCF practices. In intensive areas, a new cadre of nutrition‐focused frontline workers (Pushti Kormi), together with the health volunteer, conducted multiple age‐targeted IYCF‐focused counselling visits to households with pregnant women and mothers of children under 2 years of age, coached mothers as they tried out the practices and engaged other family members to support the behaviours. In intensive areas, CM included sensitization of community leaders to IYCF, and community theatre shows focused on IYCF. In non‐intensive areas, CM was less structured and covered general health care topics such as family planning, pregnancy registration and antenatal care and did not include IYCF‐related information. The programme evaluation design consisted of two cross‐sectional household surveys, one at baseline (2010) and 4 years later (2014) in the same communities.

**BOX 2:** PMA2020 survey and nutrition module

PMA2020 works through partnerships between a network of universities, ministries of health, national statistical agencies and research institutions across the globe to improve the way global family planning and health data are collected. The programme promotes effective data use for decision‐making by building the capacity of country partners to design and implement innovative, high‐quality, low‐cost data collection efforts that empower stakeholders to make informed policy and programme decisions. In 2017–2018, the PMA2020 programme piloted a new nutrition module in Burkina Faso and Kenya that was designed to provide government and development partners with more complete data on the coverage of nutrition‐specific interventions, diet and nutritional status among children under 5, adolescent girls and women of reproductive age, as well as household‐level food security and access to fortified foods. The new module also included a Service Delivery Point (SDP) questionnaire to assess the readiness of public and private facilities to provide nutrition services. The questionnaires were designed, tested and refined over two rounds of nationally representative data collection in each country. The PMA2020 nutrition module included questions about coverage of nutrition‐specific interventions and diet that are not commonly measured in nationally representative household surveys.

### 3.1 Timing of contact

**FIGURE 2:**
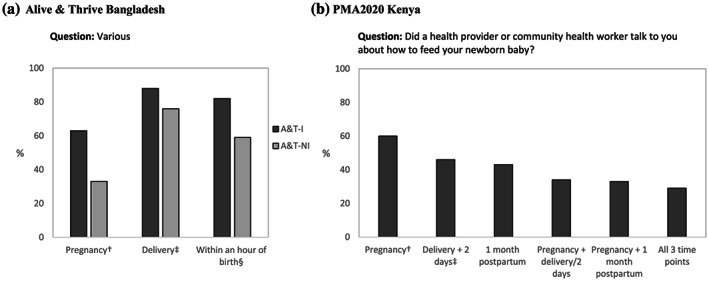
Percentage of women with children aged 0–23 months who reported receiving breastfeeding counselling at different time points. (a) Alive & Thrive Bangladesh. A&T‐I: Alive &Thrive—Intensive (*n* = 2,100); A&T‐NI: Alive & Thrive—Non‐intensive (*n* = 2,100). ^†^Those who answered ‘yes’ to the question, ‘During your last pregnancy, did you receive any counselling about breastfeeding infants and young children?’ ^‡^Those who answered ‘yes’ to the question, ‘While you gave birth to your last child, did anyone help or give advice to you regarding breastfeeding?’ ^§^Those who answered ‘within 1 hour’ to the question, ‘how many hours after your last child's birth did you first get help with breastfeeding?’ (b) PMA2020 Kenya. ^†^Among those who answered ‘yes’ to the question, ‘did you see anyone for antenatal care during your pregnancy with [child]?’ (*n* = 434). ^‡^Among those who answered any type of health facility to the question, “Where did you deliver [child]?” (*n* = 440)

The first element commonly asked about is timing of counselling contact. We identified six general time periods during which IYCF counselling occurs and are reflected in survey questions: (1) pregnancy or during ANC visits, (2) at or immediately after delivery, (3) first 6 weeks after childbirth or during PNC visits, (4) 1–5 months of age, (5) 6–23 months of age and (6) sick child contacts. Decisions around how to frame the time period in a question and/or indicator need to align with context‐specific protocols including routine health service contacts, recommended guidelines and design of the counselling intervention. For example, PMA2020 Kenya includes questions around four key time points: during ANC, at delivery or PNC (within 2 days), visit within 1 month of delivery and during a sick child contact. The early PNC (within 2 days) period was aligned to questions in the core DHS women's questionnaire. In addition to asking about each specific time point, this information can be combined to have an overall understanding of the number of key contacts mothers have regarding breastfeeding during ANC/PNC (Figure 2). Similar questions were asked in A&T Bangladesh, but with a slight difference around delivery, which excluded the first 2 days after birth (Figure 2). Another way to structure questions about timing is to ask about the ‘time elapsed’ relative to another event. In A&T Bangladesh, respondents were asked about the number of hours after birth that they first received help with breastfeeding (Figure 2). Though the majority of women answered this question, we do see a higher number of missing values compared with other question formats, alluding to potential difficulty to recall time in general, but especially around the time of a stressful event (13% in A&T‐intensive and 29% in A&T‐non‐intensive areas compared with 0% for the other formats, data not shown). There is mixed evidence on women's ability to report on care received during certain time points, especially during the immediate postnatal period (Blanc, Diaz, McCarthy, & Berdichevsky, 2016; McCarthy et al., 2016; McCarthy, Blanc, Warren, & Mdawida, 2018; Moran et al., 2013; Stanton et al., 2013).

### 3.2 Target behaviour/content

A second element consists of specifying the age‐appropriate target behaviour or the content of counselling and support received, for example, early initiation of breastfeeding and hands‐on support for proper positioning and attachment. Asking about specific content sheds light on programme fidelity and quality (Blanc et al., 2016). However, there are several considerations for how to incorporate specific counselling content in survey questions, including question content and structure.

**FIGURE 3:**
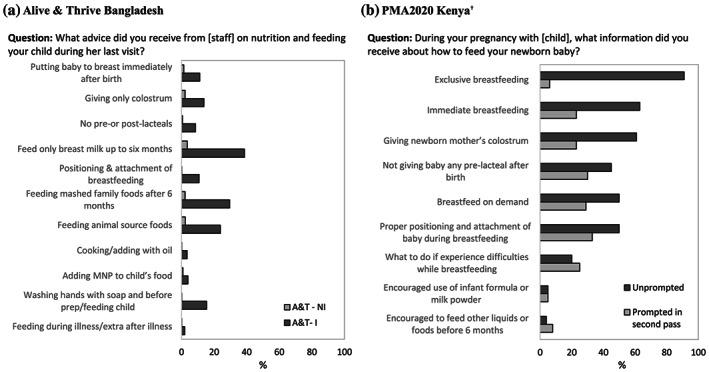
Percentage of women with children aged 0–23 months who reported receiving counselling on specific messages during last home visit. (a) Alive & Thrive Bangladesh. A&T‐I: Alive & Thrive—Intensive (*n* = 1,237); A&T‐NI: Alive & Thrive—Non‐intensive (n = 136); MNP, micronutrient powder. (b) PMA2020 Kenya. ^†^Among those who answered ‘yes’ to the question, ‘During your pregnancy with [child], did you ever receive any information from a health worker or community health worker about how to feed your newborn baby?’ (*n* = 271)

For the question about content of counselling sessions, some large‐scale national surveys may identify more general IYCF messages that are aligned with the WHO‐UNICEF IYCF recommendations (World Health Organization, 2008) or national policy documents, whereas programme‐specific survey tools designed to measure coverage of specific interventions are more likely to ask about detailed messages. For example, compared with the PMA2020 Kenya questionnaire, the response options in the A&T Bangladesh questionnaire are much more detailed and intervention specific. It includes additional topics such as feeding during illness, handwashing before food preparation and feeding, providing prelacteal/postlacteal feeds and cooking with oil. The three most commonly reported contents from IYCF counselling by respondents in A&T Bangladesh survey were exclusive breastfeeding in the first 6 months after birth, feeding mashed family foods after 6 months and the inclusion of animal source foods after 6 months (Figure 3). More specific content, such as not providing prelacteal/postlacteal feeds or cooking with oil, were reported less. One reason for higher reports of more general advice may be due to repeated exposure to the same messages from multiple interventions. Previous work has shown a tendency of women to over‐report some standard prevention practices in maternal and newborn health care, particularly related to initial assessments after delivery and immediate PNC (Blanc et al., 2016). Question structure is discussed in Section 4.2 below.

### 3.3 Place of contact

**FIGURE 4:**
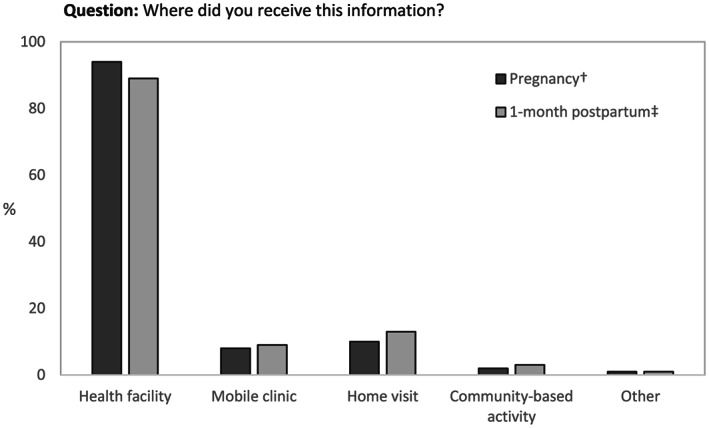
Percentage of women with children aged 0–23 months who reported receiving information from health worker about how to feed baby at specific locations during pregnancy and 1‐month postpartum, PMA2020 Kenya. ^†^Among those who answered ‘yes’ to the question, ‘During your pregnancy with child, did you ever receive any information from a health worker or community health worker about how to feed your newborn baby?’ (*n* = 271). ^‡^Among those who answered ‘yes’ to the question, ‘During child's first month of life (after delivery/first 2 days), did a health provider or community health volunteer/worker talk to you about how to feed your baby?’ (*n* = 204)

The location of counselling, such as at a health care facility or at home, is also important to consider and varies by context. Studies assessing coverage of community‐based maternal nutrition and child health interventions illustrate the importance and usefulness of including questions on the location where services are received (Blanc et al., 2016; Hazel, Requejo, David, & Bryce, 2013). This information allows researchers and programme implementers to understand where to focus resources to ensure correct messages are received. In PMA2020 Kenya, women were asked about the location where they received information about IYCF during pregnancy and at 1‐month postpartum (Figure 4). One limitation of the PMA2020 question is that differentiation of types of health care facilities (public vs. private, hospital vs. clinic) may not always be clear to women, or colloquial terms may not match survey classifications, resulting in misclassification (Carter et al., 2018). Having an open‐ended structure, or combining several questions, such as the name of the provider or additional prompting, could help verify the location of contact (Blanc et al., 2016; Carter et al., 2018) but also adds length and complexity to the questionnaire.

### 3.4 Type of service provider

The type or cadre of service providers delivering counselling ranges from health care professionals (e.g., doctors, nurses and midwives) to community volunteers and non‐governmental organization workers. Similar to location, choice of which service providers to include in response items and to code for a specific indicator will vary by context and programme design. Some contexts may require questions specific to health care workers, whereas others, particularly in rural settings, may include untrained personnel. In large‐scale national surveys such as PMA2020 Kenya, a more detailed response list can be too time‐consuming, so questions often have discrete response options for different professional staff, and then combine non‐professional staff into a single response category (Table 1). Similar to place of contact, collecting data on the type of service provider who provided counselling is not always straightforward. First, women may have difficulty discerning between different types of providers (Adegoke, Utz, Msuya, & van den Broek, 2012; Blanc et al., 2016). Second, recall bias may be likely for reporting of stressful events. In a validation study in Mozambique, women were able to accurately report the presence of a support person during labour and delivery (Stanton et al., 2013); however, this was not the case in studies from other contexts (Blanc et al., 2016). Third, concern over negative perceptions may cause under‐reporting of the use of traditional practitioners in certain contexts (Carter et al., 2018; King, 2012; Muula, Polycarpe, Job, Siziya, & Rudatsikira, 2009). Women may not consider treatment by a traditional practitioner as ‘seeking care’ (Carter et al., 2018). These issues could be addressed and clarified in survey pretesting.

**TABLE 1:** Percentage of women with children aged 0–23 months who received breastfeeding counselling from different service providers during pregnancy, delivery (Alive & Thrive Bangladesh) and 1‐month postpartum (PMA2020 Kenya)

Question: Who did you receive this counselling from?			
	A&T‐I	A&T‐NI
During pregnancy:	*n* = 1,330^a^ (%)	*n* = 655^a^ (%)
Doctor/clinical officer/nurse/midwife	16.7	39.7
Government health worker	3.4	10.7
Shasthya Shebika	51.2	12.2
Shasthya Kormi	13.1	10.8
Pushti Kormi	8.0	0.0
Other NGO worker	0.5	5.0
TTBA\TBA\village doctor	1.1	4.7
Family members	5.7	16.1
At delivery:	*n* = 1,849^b^ (%)	*n* = 1,593^b^ (%)
Doctor/clinical officer/nurse/midwife	21.9	21.7
Government health worker	0.2	0.8
Shasthya Shebika	2.9	1.0
Shasthya Kormi	0.7	0.4
Pushti Kormi	0.4	0.0
Other NGO worker	0	0.2
TTBA\TBA\village doctor	19.8	12.7
Family members	51.9	62.6
Question: During child's first month of life (after delivery/first 2 days) which health provider or community health volunteer/worker talked to you and/or observed your breastfeeding?			
	PMA2020 Kenya		
1‐month postpartum:	*n* = 237^c^ (%)		
Doctor/clinical officer	25.0		
Nurse/midwife	72.0		
Traditional birth attendant	8.0		
Community health volunteer/worker	8.0		
Other	5.0		
No response	1.0		

Abbreviations: A&T–I, Alive &Thrive–—Intensive; A&T‐NI, Alive & Thrive—Non‐intensive; TBA, traditional birth attendant; TTBA, trained traditional birth attendant.

a Those who answered ‘yes’ to the question, ‘During your last pregnancy, did you receive any counselling about breastfeeding infants and young children?’

b Those who answered ‘yes’ to the question, ‘While you gave birth to your last child, did anyone help or give advice to you regarding breastfeeding?’

c Those who answered ‘yes’ to the question, ‘During child's first month of life (after delivery/first 2 days), did a health provider or community health volunteer/worker talk to you about how to feed your baby?’

### 3.5 Frequency

**FIGURE 5:**
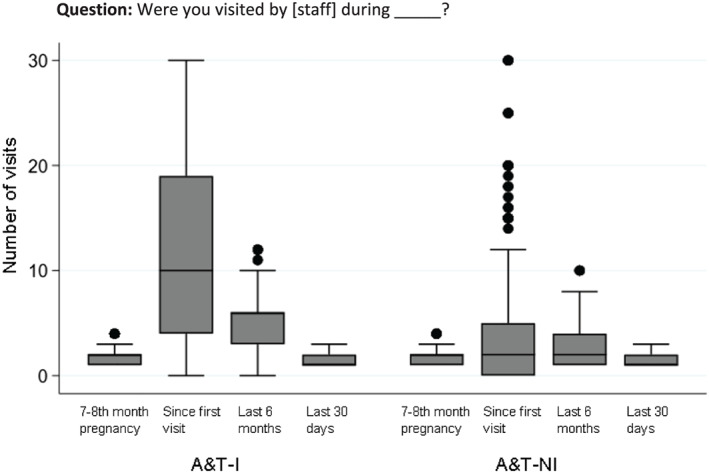
Frequency of health visits to women with children ages 0–23 months by community health volunteer across different recall periods, Alive & Thrive Bangladesh. A&T‐I: Alive & Thrive—Intensive (*n* = 2,100); A&T‐NI: Alive & Thrive—Non‐intensive (*n* = 2,100)

The frequency of counselling relates to whether any counselling occurred, the number of sessions and/or the duration of session received. Asking about frequency helps assess programme fidelity in contexts where multiple counselling contacts are intended. It can also help examine the association between the frequency of contact and targeted behaviours and help determine how many visits are adequate. Although substantial evidence exist on the number of contacts during ANC (Hodgins & D'Agostino, 2014; Moran et al., 2016), there is a paucity in data on frequency of visits during the postnatal period (Moran et al., 2013). We demonstrate the different ways in which survey questions try to capture frequency of visits from A&T Bangladesh (Figure 5) and discuss some considerations for each. Longer, unbound recall periods such as visits after giving birth and in the last 6 months have a larger spread of responses and more outliers. Conversely, shorter, more specific recall periods like visits during the 7–8 months pregnancy period and in the last 30 days have less spread of data points but may not be appropriate to assess coverage if contacts are expected to be less frequently than monthly. Thus, the recall period and exact timing for asking about frequency may depend on the programme design. Another common question on frequency refers to ‘any/at least one’ visit. Although this is easier to tabulate, the binary nature of the resulting data does not provide much information beyond knowing if any visit has happened. It is therefore important to consider when it is useful to use this one‐time frequency versus a more detailed set of questions, as well as the trade‐offs with survey length and potential recall bias.

### 3.6 Mode of intervention

The mode of counselling includes individual or one‐to‐one contact, group sessions and via technology (mobile devices or internet). Differentiating the mode of interventions used is important in programme evaluation contexts because they often differ in terms of scope and level of detail discussed. For programmes, it can also help to assess the impact (individual, additive or multiplicative) of having a single or combined intervention (Menon, Nguyen, Saha, Khaled, Kennedy, et al., 2016). Including detailed response options may help women differentiate modes. Women may also perceive the various modes, or even the concept of counselling itself, differently across contexts. For example, a study reported that women did not understand what was meant by a PNC contact defined as a ‘health check‐up’ or ‘a check on your health’ after the baby was born, unless study teams defined the term (Yoder et al., 2010). In Malawi, interviewers provided examples of PNC content; in Bangladesh, interviewers described what was meant by health in general (Yoder et al., 2010). Similar to other elements of counselling coverage, deciding on the level of detail to include in response options should be based on programme design and the local context and in line with local understanding of the different modes of counselling.

## 4 CROSS‐CUTTING ISSUES IN DEVELOPING SURVEY QUESTIONS

There are several considerations for question design, which cut across all the elements discussed above. We discuss three main considerations: recall period, question structure (prompted vs. unprompted) and internal validity. Other considerations that are important but not discussed in this paper include the mode of data collection [paper‐based vs. computer‐assisted personal interviewing (CAPI)] (Zeleke, Naziyok, Fritz, & Röhrig, 2019) and determining the right balance between survey length, detail and the implications for resources and potential survey fatigue (Olson, Smyth, & Ganshert, 2019).

### 4.1 Recall period

The recall period pertains to the length of time respondents are asked to consider in responding to a question about when they received counselling. Recall periods for reporting counselling received vary in length and detail. The periods could reference specific life stage (e.g., pregnancy), time of contact (e.g., in the first month after child was born) or duration of recall, which respondents are asked to remember (e.g., in the last month). Common durations in questionnaires included wider recall periods, like the last 6 months and the last year, whereas specific recall periods or timings included the last 7–8 months of pregnancy, in the first 2 days after delivery or during the last visit with a health care provider. The choice and presentation of recall period can influence the accuracy of reporting (Hazir et al., 2013; McCarthy et al., 2018). An example of different ways of asking about recall periods from PMA2020 Kenya can be found in Table S3. In 2017, different recall periods were specified in the question stem according to the child's current age: caregivers of children 0 to 5 months were asked if they *ever* received advice; caregivers of children 6 to 11 months were asked if they received advice in *the last 1 month*; and caregivers of children 12–23 months were asked if they received advice in *the last 3 months.* However, this made it difficult to compare findings across age groups. In 2018, recall period was not specified in the stem of the question. Instead, caregivers of all children 0–23 months of age were asked a follow‐up question about the number of months elapsed since they last received advice about child feeding. The 2018 data showed that elapsed time since last counselling contact for older children was longer compared with caregivers of younger children: 67% of caregivers with children 0–5 months old reported receiving advice on child feeding less than 1 month ago compared with only 46% of 6–11 months old and 26% of 12–23 months old. However, 49% of caregivers with children 6–11 months old reported receiving advice in the past 1–11 months, and 50% with children 12–23 months old reported receiving advice in the past 1–11 months. This is expected as IYCF counselling tends to occur most intensively during the period of recommended exclusive breastfeeding and in early transition to complementary foods around 6 months of age. Besides recall periods as number of months, asking about specific time frames, for example the 7th to 8th month of pregnancy, may be easier to recall with accuracy compared with a vague time frame such as the last time a visit occurred (if this visit was not recent). The more salient the intervention or event, the more accurately women can recall the event over time, even at 13–15 month follow‐up (McCarthy et al., 2016).

Insights on recall period are found in other health topics. Increasing the recall period of childhood pneumonia from 2 to 4 weeks (Hazir et al., 2013) or even 8 weeks (Feikin et al., 2010) did not affect the accuracy of mothers' reports. One paper suggests that survey timing relative to the period of peak incidence should also influence choice of recall period (Campbell et al., 2013). A study in Kenya on reporting symptoms of cough, fever, and diarrhoea and health‐seeking behaviour found that a 2‐week recall period underestimated true disease and health care utilization rates and suggested using shorter recall periods of 3–4 days to obtain more accurate data (Feikin et al., 2010). One potential reason for the difference between these two examples is that a severe or traumatic event like child pneumonia could be less subject to recall bias compared with something more common like cough or fever. A study in Guatemala assessing mothers' recall of child symptoms of illness occurring in the previous 2 weeks found that an important aspect of reporting was the duration of symptoms: the longer the duration of the symptom, the greater the probability of it being reported by mothers (Martorell, Habicht, Yarbrough, Lechtig, & Klein, 1976). Although there was a slight difference in reporting based on severity of the symptoms, the range of severity in the study was very narrow, making it difficult to draw conclusions on the effect of symptom severity on recall (Martorell, Habicht, Yarbrough, Lechtig, & Klein, 1976). In relation to childhood malaria and insecticide‐treated bed net coverage, a study in Zambia found no drop‐off in the accuracy of recalling malaria diagnosis and treatment interventions 2 versus 1 week after the clinic visit (Eisele et al., 2013), but did find substantial recall bias and date heaping at 12 months for recall of when a bed net was procured or treated (Eisele, Macintyre, Yukich, & Ghebremeskel, 2006).

Only a few studies on IYCF practice measurement explored the accuracy of different recall periods. Bland et al. (2003) suggested that long‐term recall data on exclusive breastfeeding are inaccurate and recommended prospective data collection of exclusive breastfeeding at intervals of no longer than 1 week. On the other hand, a review examining the validity and reliability of maternal recall of breastfeeding history observed that maternal recall is a valid and reliable estimate of breastfeeding initiation and duration, especially when the duration of breastfeeding is recalled under 3 years; this was not the case for maternal recall for the age at introduction of complementary foods (Li, Scanlon, & Serdula, 2005). In order to address recall bias, it may be useful to use a range of recall periods into the survey to address recall decay and also to determine the optimal recall period for a specific intervention (Munos et al., 2018).

### 4.2 Prompted versus unprompted questions

The way a question is asked by the interviewer influences what is reported. In PMA2020 Kenya, a question about content of breastfeeding messages during ANC counselling was first asked without prompts, to allow respondents to freely list the messages they received (unprompted). Then, if there were any response options not mentioned, the enumerator will ask about those specifically (prompted). We see large variations in the results by topic for unprompted responses and after prompting (Table S4). When deciding on whether to include prompted or unprompted questions around content, it is important to consider some limitations of prompted questions. As more programmes become multisectoral in nature and include an increasing number of topics, prompted questions on content discussed in counselling may have time and cost implications, as well as lead to fatigue for both the enumerator and respondent. Moreover, recall has been shown to be influenced by the order of response options provided in prompted or close ended questions (Lunet et al., 2008).

### 4.3 Internal validity

Previous work suggests that when self‐reported data are used, multiple questions regarding when and how the intervention was received should be used in order to triangulate findings to enhance internal validity (McCarthy et al., 2016). For example, by combining questions on content and timing, we can understand the quality and appropriateness of counselling. One would expect higher reporting of early initiation of and exclusive breastfeeding messages during pregnancy, higher reporting of messages about how to address breastfeeding problems in the first month after birth and higher reporting of complementary feeding messages around 6 months, compared with other time points. Combining information in this way sheds light on the appropriateness of the timing of the messages and women's ability to differentiate the messages received.

Another example of triangulating responses is combining questions around service provider and specific time points. For example, from A&T Bangladesh data, mothers reported frontline health workers mostly provided counselling during ANC, whereas at delivery, doctors and family members provided counselling (Table 1 **)**. This is an important information to have because different services might be delivered by different providers and content and modes of providing information may vary depending on who conducts counselling.

A third example of triangulating questions to increase internal validity is the case of A&T Bangladesh where a series of questions was asked to see whether mothers could differentiate between the different types of service providers. With the example of the community health volunteer, we observed that 74.9% and 33.2% of women in intensive and non‐intensive arms, respectively, reported knowing who the health volunteer was in their area by title/name. However, after seeing a photo of the health volunteer, substantially more women reported recognizing the volunteer compared with those who initially said they knew the person. This indicates that some respondents may not be aware of the health volunteer's title; other studies have also shown that respondents have difficulty in identifying or differentiating between different service providers (Adegoke et al., 2012; Blanc et al., 2016).

## 5 CONCLUSION

There is growing recognition of the importance of evidence‐based indicators and protocols for collecting data on the coverage of health interventions (Moran et al., 2013) as well as on the reach of nutrition interventions (Gillespie et al., 2019). Making progress on coverage measurement requires harmonization and alignment across a range of actors supporting, implementing and evaluating programmes. The lack of data to track coverage of counselling interventions was highlighted as a major gap in nutrition coverage measurement (Amouzou et al., 2019). This paper, drawing on a consultative process involving diverse actors in the international nutrition community, provides an opportunity to ensure better IYCF counselling coverage data. Applying the proposed framework on the six elements of counselling coverage questions together may help to guide IYCF counselling coverage measurement and indicator development. This in turn may allow for better tracking of progress towards providing caregivers of infants and young children with age‐appropriate counselling services. Further research on testing and validating the coverage questions is needed to strengthen evidence for a set of valid and reliable coverage indicators. Such indicators will help to identify bottlenecks in IYCF counselling implementation. Future research should also consider harmonizing and triangulating survey‐based coverage data with local administrative data, for which standardization of indicators is currently underway.

## CONFLICTS OF INTEREST

The authors declare that they have no conflicts of interest. KKS and LGS are members of the World Health Organization. The authors alone are responsible for the views expressed in this publication and they do not necessarily represent the decisions, policy or views of the World Health Organization.

## CONTRIBUTIONS

JC, SSK, PHN and RH conceptualized this paper; JC and PN supported in data analyses; JC and SSK drafted the paper; and PN, RH, LGS, KKS, CH, VM, SA and PM provided technical inputs to the drafts. All authors read and approved the final manuscript.

## Supporting information


**Table S1.** Survey questions recommended by the Joint Consultation on Approaches to Measure Coverage of Nutrition Counselling Interventions
**Table S2.** List of studies or surveys included in the review
**Table S3.** Examples of questions on complementary feeding counselling that differed in child age and recall periods across two rounds of the PMA2020 Kenya survey (2017 and 2018)
**Table S4.** Percentage of women with children aged 0–23 months who reported receiving specific breastfeeding messages during counselling, PMA2020 Kenya
