# Supplementary material for: Measuring coverage of infant and young child feeding counselling interventions: A framework and empirical considerations for survey question design
Source: Matern Child Nutr. 2020 Apr 15;16(4):e13001. doi: 10.1111/mcn.13001 (PMC7507318; doi:10.1111/mcn.13001)
Supplement: Supplementary file 1 — Table S1. Survey questions recommended by the Joint Consultation on Approaches to Measure Coverage of Nutrition Counselling Interventions Table S2. List of studies or surveys included in the review Table S3. Examples of questions on complementary feeding counselling that differed in child age and recall periods across two rounds of the PMA2020 Kenya survey (2017 and 2018) Table S4. Percentage of women with children aged 0–23 months who reported receiving specific breastfeeding messages during counselling, PMA2020 Kenya [file MCN-16-e13001-s001.docx]

**Supplementary Table 1. Survey questions recommended by the Joint Consultation on Approaches to Measure Coverage of Nutrition Counseling Interventions**

| **No.** | **Question** | **Response** |
| --- | --- | --- |
| 1 | During this pregnancy, did a health care provider or community worker talk with you about  breastfeeding?  (*to be asked with other antenatal care questions*) | ⃝ Yes  ⃝ No  ⃝ Don’t Know |
| 2 | During the first two days after (NAME)’s birth, did any health care provider do the following:  (*to be asked with other postnatal care questions*) | ⃝ Examine the chord?  ⃝ Measure Temperature?  ⃝ Counsel you on danger signs for newborns?  ⃝ Counsel you on breastfeeding?  ⃝ Observe breastfeeding? |
| 3 | During the first month after (NAME)’s birth (but after first two days), did a health care provider or community worker talk with you about breastfeeding?  (*to be asked with other postnatal care questions*) | ⃝ Yes  ⃝ No  ⃝ Don’t Know |
| 4 | In the last six months, did a health care provider or community worker talk with you about how to feed your child? | ⃝ Yes  ⃝ No  ⃝ Don’t Know  ⃝ No response |
| 4a | (If yes) What topics did he or she talk with you about?  (*list would differentiate topics on breastfeeding and topics on complementary feeding*) | <To be determined> |

**Supplementary Table 2. List of studies or surveys included in the review**

| Project/survey | Country | Survey round |
| --- | --- | --- |
| Program evaluations | | |
| Alive & Thrive (A&T), IYCF study | Bangladesh | Endline 2014 |
|  | Vietnam | Endline 2014 |
|  | Ethiopia | Endline 2017 |
|  | Burkina Faso | Endline 2017 |
| Alive & Thrive (A&T), Maternal nutrition study | Bangladesh | Endline 2016 |
|  | India | Baseline 2017 |
| Common Application Software (CAS) | India | Process Evaluation 2017 |
| Convergence | India | 2014 |
| Mama SASHA | Kenya | Endline 2014 |
| Preventing Malnutrition in Children under 2 Approach (PM2A) | Guatemala, Burundi | Endline 2014 |
| Innovative Approaches for the Prevention of Undernutrition (PROMIS) | Burkina Faso, Mali | Endline 2017 |
| Soutenir l’Exploitation Familiale pour Lancer l’Élevage des Volailles et Valoriser l’Économie Rurale (SELEVER) | Burkina Faso | Endline 2017 |
| Sanitation Hygiene Infant Nutrition Efficacy trial (SHINE) | Zimbabwe | Baseline 2016 – 18 month visit |
| Transfer Modality Research Initiative (TMRI) | Bangladesh | Endline 2014 |
| Targeting and Realigning Agriculture for Improved Nutrition (TRAIN) | Bangladesh | Baseline 2016 |
| Women Improving Nutrition through Group-based Strategies (WINGS) | India | Midline 2017 |
| Nationally representative surveys | | |
| Performance Monitoring and Accountability 2020 (PMA2020) | Burkina Faso, Kenya | Round 2 2018 |
| Demographic and Health Survey (DHS) - 7 | Various |  |
| Multiple Indicator Cluster Survey (MICS) - 6 | Various |  |

**Supplementary Table 3. Examples of questions on complementary feeding counseling that differed in child age and recall periods across two rounds of the PMA2020 Kenya (2017 and 2018)**

|  | **2017** | |
| --- | --- | --- |
| 1 | ***(Mothers with children aged 0-5 months)***  Did you ever receive any advice from a health worker at a facility about what liquids, semi-solid and solid foods to provide your child, other than breastmilk? | ⃝ Yes  ⃝ No  ⃝ Don’t Know  ⃝ No response |
| 2 | ***(Mothers with children aged 6-11 months)***  In the last 30 days, did you receive any advice from a health provider at a facility about what liquids, semi-solid, and solid foods to provide your child, other than breastmilk? | ⃝ Yes  ⃝ No  ⃝ Don’t Know  ⃝ No response |
| 3 | ***(Mothers with children aged 12-23 months)***  In the last 3 months, did you receive any advice from a health provider at a facility about what liquids, semi-solid, and solid foods to provide your child, other than breastmilk? | ⃝ Yes  ⃝ No  ⃝ Don’t Know  ⃝ No response |
|  | **2018** | |
| 1 | ***(All mothers)***  Have you ever received any advice from a health provider or community health volunteer/worker about what liquids, semi-solid and solid foods to provide your child, other than breastmilk? | ⃝ Yes  ⃝ No  ⃝ Don’t Know  ⃝ No response |
| 2 | How long has it been since you last received advice on feeding your child from a health provider or community health volunteer/worker? | ⃝ Less than 1 month  ⃝ 1–11 months  ⃝ 1 year or more  ⃝ Don’t Know  ⃝ No response |

**Supplementary Table 4. Percentage of women with children aged 0-23 months who reported receiving specific breastfeeding messages during counseling, PMA2020 Kenya**

| **Question:** During your pregnancy with [child], what information did you receive about how to feed your newborn baby? | | |
| --- | --- | --- |
|  | **Unprompted**  **(n=271)^†^ (%)** | **Prompted in second pass**  **(n=271)^†^ (%)** |
| Exclusive breastfeeding (only feeding breast milk and nothing else except for prescribed medicine; no water) | 91 | 6 |
| Immediate breastfeeding (putting baby to breast immediately after birth/within 1 hour) | 63 | 23 |
| Giving newborn mother’s colostrum (first yellowish milk) | 61 | 23 |
| Not giving baby any water, sugar water, tea or traditional preparation after birth | 45 | 30 |
| Breastfeed on demand (feeding whenever baby wants/signals) | 50 | 29 |
| Proper positioning and attachment of baby during breastfeeding (show you how to hold your baby while breastfeeding) | 50 | 33 |
| What to do if experience pain or problems while breastfeeding | 20 | 25 |
| Encouraged use of infant formula or milk powder | 5 | 5 |
| Encouraged to feed other liquids or foods before 6 months | 4 | 8 |
| None of the above | 0 | 16 |
| No response | 0 | 0 |

^†^Those who answered “yes” to the question, “During your pregnancy with [child], did you ever receive any
 information from a health worker or community health worker about how to feed your newborn baby?”
